# Supplementary material for: Discrimination and Characterization of the Volatile Organic Compounds in Schizonepetae Spica from Six Regions of China Using HS-GC-IMS and HS-SPME-GC-MS
Source: Molecules. 2022 Jul 8;27(14):4393. doi: 10.3390/molecules27144393 (PMC9319859; doi:10.3390/molecules27144393)
Supplement: Supplementary file 1 [file molecules-27-04393-s001.zip › Table S1. Detailed information of all the samples.pdf]

Table S1. Detailed information of all the samples.

Number	Origin	Batch number
B1	Hebei	1905011
B2	Hebei	2012002
B3	Hebei	201101CP171
N1	Henan	2020107
N2	Henan	2020106
N3	Henan	191005
S1	Shandong	202108010
S2	Shandong	20211010
S3	Shandong	2021018
J1	Jiangsu	200301
J2	Jiangsu	190701
J3	Jiangsu	190801
A1	Anhui	20211112
A2	Anhui	20211107
A3	Anhui	2020105
Z1	Zhejiang	190501
Z2	Zhejiang	1210808
Z3	Zhejiang	190601
